# Identifying nursing sensitive indicators from electronic health records in acute cardiac care―Towards intelligent automated assessment of care quality

**DOI:** 10.1111/jonm.13802

**Published:** 2022-09-27

**Authors:** Hanna von Gerich, Hans Moen, Laura‐Maria Peltonen

**Affiliations:** ^1^ Turku University Hospital, Department of Nursing Science University of Turku Turku Finland; ^2^ Department of Computer Science Aalto University Espoo Finland; ^3^ Department of Nursing Science University of Turku Turku Finland

**Keywords:** acute cardiac care, care quality assessment, electronic health records, nursing care quality, nursing‐sensitive indicators

## Abstract

**Aim:**

The aim of this study is to explore the potential of using electronic health records for assessment of nursing care quality through nursing‐sensitive indicators in acute cardiac care.

**Background:**

Nursing care quality is a multifaceted phenomenon, making a holistic assessment of it difficult. Quality assessment systems in acute cardiac care units could benefit from big data‐based solutions that automatically extract and help interpret data from electronic health records.

**Methods:**

This is a deductive descriptive study that followed the theory of value‐added analysis. A random sample from electronic health records of 230 patients was analysed for selected indicators. The data included documentation in structured and free‐text format.

**Results:**

One thousand six hundred seventy‐six expressions were extracted and divided into (1) established and (2) unestablished expressions, providing positive, neutral and negative descriptions related to care quality.

**Conclusions:**

Electronic health records provide a potential source of information for information systems to support assessment of care quality. More research is warranted to develop, test and evaluate the effectiveness of such tools in practice.

**Implications for Nursing Management:**

Knowledge‐based health care management would benefit from the development and implementation of advanced information systems, which use continuously generated already available real‐time big data for improved data access and interpretation to better support nursing management in quality assessment.

## 1 BACKGROUND

Providing high‐quality health services is key in managing cardiovascular diseases and improving patient outcomes (Thomas et al., 2018). Despite the efforts in achieving a downward trend of disease prevalence in high‐income countries (Amini et al., 2017), cardiovascular diseases are still an important cause of mortality globally, causing over 38% of premature deaths in 2019 (WHO, 2021). Improving service quality has been estimated to prevent up to 2.5 million of these deaths annually (WHO, 2020a). Acquiring good quality is a continuous cycle of planning, implementing and evaluating quality improvement activities in all levels of a health care system (WHO, 2020b). On the clinical level, systematic quality assessment of cardiac care using illness‐specific quality metrics has the potential to improve care outcomes, but to be effective, the results should be connected to patient‐related outcomes, such as patient experience or mortality (Chatterjee & Joynt, 2014). The use of nursing‐sensitive indicators could provide a more holistic perspective, focusing on the assessment of nursing care quality (Heslop et al., 2014). However, previous literature shows little evidence of the application of these indicators in the assessment of cardiac care.

The use of nursing‐sensitive indicators can help build a foundation to quantify, measure and improve dynamic nursing care quality within all domains of nursing (Afanef et al., 2021). In fact, systematic assessment of quality based on nursing‐sensitive indicators have shown to improve the quality of nursing care (Elgseer et al., 2021). For example, nurse‐to‐patient ratios in acute care have been proven to influence a variety of patient outcomes, such as mortality, providing valuable information when constructing optimal staffing models (Driscoll et al., 2018). In cardiac care, the use of indicators in monitoring the quality of highly specialized procedures, such as cardiac catheterization, has an impact on their quality and safety (Shen et al., 2021). However, issues regarding the selection of appropriate indicators to use, report and embed in clinical practice hinder their introduction in continuous quality assessment and improvement initiatives (Burston et al., 2013). A recent study indicated that barriers to quality improvement for nurse managers included a lack of timely data presented in a usable and easy‐to‐access manner (Alexander et al., 2022). Additionally, information needs vary between different actors and settings in health care, such as professionals, units and work shifts, creating a need for tailored and dynamic information systems, which respond to different users' individual needs (Peltonen et al., 2019). From a nurse's standpoint, providing requisite data entries for quality assessment generally increases documentation time and results in a need to make duplicate entries to differing documentation systems (Elgseer et al., 2021).

The potential of using electronic health records (EHRs) as a secondary data source for quality assessment has been recognized since their introduction. From early on, concerns regarding the accurate portrayal of the complexity of care and data requirements for comprehensive quality assessment as well as accuracy, comparability and timeliness of extracted data have been presented (Roth et al., 2009). These issues are as relevant today, as the use of single nursing‐sensitive indicators extracted from EHRs to assess nursing care quality is on the increase. One example is the automatic detection of medication errors by comparing medication prescribing and documentation as extracted from EHRs (Kirkendal et al., 2020).

Research utilizing multiple nursing‐sensitive indicators simultaneously to form a holistic picture of care quality in acute cardiac care is lacking to our knowledge. In the intensive care setting, efforts to use EHR data when manually extracting information on care quality are showing encouraging results. A study by Seaman et al. (2017) showed that combining structured and free text entries describing selected quality outcome measures provided accurate information on quality. The included measures were heavy sedation, use of physical restraints, presence and intensity of pain, unplanned extubation and pressure injuries. The used manual extraction tool in the study was considered labour‐intensive, and the use of free text notes prohibited the use of automated extraction methods available to the researchers (Seaman et al., 2017).

Compared with manual extraction methods, automated methods to extract quality information from EHRs show advantages in data integrity, reliability and accuracy (Brundin‐Mather et al., 2018). Introducing artificial intelligence (AI), a range of intelligent technologies present the best possible solutions in automated problem detection or prediction to improve safe patient care (Sensmeier, 2017). Methods utilizing AI have proven their applicability. Machine learning approaches, for example, hold the potential to be used in identifying inpatient fall risks from EHR's and administrative data (Lindberg et al., 2020). Natural language processing (NLP), in turn, has been applied as a novel way to process and present information gathered from free text EHR notes efficiently (Juhn & Liu, 2020; Koleck et al., 2019). All in all, the use of AI in nursing‐relevant tasks is on the rise, with over 50% of said technologies using EHRs as the data source and approximately 10% of these technologies focusing on NLP (von Gerich et al., 2022).

Previous research findings indicate that quality assessment initiatives could benefit from the rich data EHRs have to offer and the use of carefully selected nursing‐sensitive indicators. This study addresses the gap of knowledge in using nursing‐sensitive indicators in assessing care quality in acute cardiac care units using EHRs as a data source. The results support the application of NLP methods on free text narratives, which could have a potential impact on effective and automated real time care quality assessment. The aim of this study was to examine the potential of using EHRs in assessing care quality through selected nursing‐sensitive indicators in acute cardiac care. The study question was: What expressions indicating nursing care quality can be identified and extracted from structured and free‐text notes in patients' EHRs?

## 2 METHODS

### 2.1 Research design

This retrospective descriptive study was guided by the theory of value‐adding analysis described by Eakin and Gladstone (2020). Value‐adding analysis is a form of qualitative research, in which conventional qualitative analysis methods are complemented by a process of analysis aiming to construct concepts on a more abstract level. The analysis held four interrelated features: interpretation, contextualization, “creative presence of the researcher” and critical inquiry. The qualitative methods used included deductive and inductive (Elo & Kyngäs, 2008) as well as summative content analysis (Hsieh & Shannon, 2005) aiming to describe and quantify the phenomena of interest. Reporting was conducted according to the COREQ‐checklist for qualitative studies (Tong et al., 2007).

### 2.2 Setting

The EHR data used in this study were collected from a cardiac centre of one out of 21 hospital districts in Finland. This cardiac centre is part of highly specialized medical care offered at public hospitals. The centre operates in a university hospital, performing acute care including cardiac and lung surgeries and day surgeries, as well as outpatient follow‐ups. In 2021, the centre had 20,562 outpatient visits and a total of 4963 in‐hospital care episodes.

The EHR system used in the cardiac centre is a multi‐professional system developed for documenting all relevant perspectives related to a patient's clinical care, including, for example, nursing and physician notes, prescriptions and treatment charts, as well as laboratory examinations and results from each service event, where the provider has interacted with a patient for care or medical treatment. Following the Finnish decree on patient records (298/2009) and the decree on patient status and rights (758/1992), all necessary and comprehensive information regarding the arrangement, planning, execution and monitoring of good patient care need to be documented in this EHR system. This information includes preliminary information regarding the patient's health status, illnesses or injuries, observations made by the caretakers, laboratory and imaging results as well as care measures to restore or maintain the patient's health or alleviate suffering. Entries made in the EHRs must be clear and understandable, containing only generally established concepts and abbreviations.

The documentation in the EHRs follows a title structure where predefined classifications are complemented using narrative free text. All health care professionals make entries to the system following codes and standards specific to their own professional groups. For nurses, the system allows to document patient care systematically by phase of nursing process. Structured data elements, such as nursing diagnosis, interventions, outcomes, intensity and discharge summary, follow the Finnish Care Classification (FinCC) system as presented by the Finnish institute for health and welfare (Kinnunen et al., 2021).

The documentation entries by different health care professionals form together an entity describing the patient's health situation and clinical pathway in a holistic and multi‐professional way. Hence, when evaluating patient care, all entries made in the EHR‐system are essential when seeking for an understanding of the overall picture.

### 2.3 Participants

The data were collected from patients (*n* = 1852) admitted and treated at the acute cardiac care units of the cardiac centre during January 2020. The data collection point was selected to ensure the most recent data but to avert the possible skewness caused by the global outbreak of COVID‐19. The data included all clinical care entries made in the centre, such as free text nursing and physician notes as well as structured clinical measurements, patient evaluations and laboratory results from all units. Only operating room reports, intensive care nursing narratives and radiology images from the cardiac centre were excluded.

The sample consisted of the EHRs of 230 patients. To ensure as wide, versatile and reliable picture as possible regarding patient care, all available entries made in the EHR were included in this study. Only including one source of the documentation, such as the nursing narratives alone, would not provide a comprehensive picture of the care provided, as the documentation in the EHR is done in a multi‐professional way with all different perspectives complementing each other. The included records held 540 care episodes lasting from one to 13 days. They contained 6867 notes, including but not limited to nursing notes (*n* = 2673) and measurements (*n* = 2336), physician notes (*n* = 527), diagnostic notes (*n* = 377) and laboratory results (*n* = 347).

### 2.4 Data collection

The data extraction from the EHR archives was performed by a computer scientist. The data were randomly organized by patient text files. Using a systematic random sampling method with a periodic interval, every fifth text file was selected until no relevant new discoveries were made (Grove, 2017). The point of saturation was determined by following a deductive codebook approach (Kerr et al., 2010), and saturation was reached after 180 patients. The saturation was verified by continuing the analysis with an additional 60 patients' records, and no new expressions were identified.

### 2.5 Data analysis

The structure of the deductive analysis was operationalized by combining selected and well‐established nursing‐sensitive indicators used for assessing care quality suitable for the acute cardiac care unit environment. These patient outcome indicators included the National Database of Nursing Quality Indicators (Montalvo, 2007) and nursing‐sensitive indicators applied to the context of acute care (Heslop et al., 2014) and served as a codebook to guide the deductive analysis process.

Expressions related to nursing care quality, such as words and word pairs, were extracted from the EHRs to a spreadsheet containing a codebook presented as a structured categorization matrix. The expressions were collected and coded by one researcher, with the results analysed and discussed together with another researcher during and after the whole data analysis process. The expressions inside the codebook categories were further divided into subcategories. They were also quantified in attempts to contextualize the context as well as to further examine the use of the expressions (Hsieh & Shannon, 2005).

### 2.6 Ethical considerations

This study utilized EHRs, which are classified as personal data. In Finland, the use of health record data in scientific research is regulated by Regulation (EU) 2016/679 of the European Parliament and of the Council, the Data Protection Act (1050/2018) and the Finnish decree on patient records (298/2009). The EHR data used in this study were pseudonymized, and patients or hospital units could not be identified from the results. The data were securely stored on, and accessed through, the servers of the hospital district. The data were managed following a confidentiality agreement.

This study followed The European Code of Conduct for Research Integrity *‐*guidelines (All European Academies, 2017). The study was a part of Smart Health Care Management ‐project of the University of Turku. The project held an ethical approval statement (9/2020) issued by the University of Turku Ethics Committee for Human sciences (Health Care Division). It was also granted an administrative approval (J14/20) by the hospital district.

## 3 RESULTS

The sample of patients consisted of 98 (43%) women and 132 (57%) men born between 1923 and 1995, ages ranging from 25 to 97 years (standard deviation 41 years). In total, 1676 expressions related to care quality were extracted of which 1235 (73.7%) originated from free text and 441 (26.3%) from structured notes.

The value‐adding analysis was concretized as an interpretative inductive analysis. The expressions were divided into two categories: *established expressions related to nursing care quality* and *unestablished expressions related to nursing care quality*. Established expressions provided straightforward information directly related to nursing care quality that could be utilized as is or with minor adjustments in a tool assessing care quality. Unestablished expressions, in turn, provided information indirectly related to nursing care quality of which more research is still needed before its application in care quality assessment. Table 1 illustrates quantities of expressions related to nursing care quality extracted from free text and structured notes.

**TABLE 1:** Expressions related to nursing care quality extracted from free text and structured notes

Expressions related to nursing care quality *n* = 1676 (100%)	Subcategory	Free text		Structured notes	
		*n*	(%)	*n*	(%)
Established expressions related to nursing care quality *n* = 748 (44.6%)	Experience of pain	458	(27.3)	204	(12.2)
	Adverse event	58	(3.5)	1	(0.1)
	Patient satisfaction	27	(1.6)	0	(0.0)
Unestablished expressions related to nursing care quality *n* = 928 (55.4%)	Physical health	124	(7.4)	235	(14.0)
	Perceived health	327	(19.5)	0	(0.0)
	Psychosocial health	185	(11.0)	1	(0.1)
	Functional health	56	(3.3)	0	(0.0)

Additionally, the value adding analysis resulted in dividing all expressions extracted from the EHR's into one of the following three categories: *positive descriptions*, *neutral descriptions* and *negative descriptions* of patient status related to nursing care quality. These categories described the informational value of the extracted expressions, distinguishing between negative, neutral and positive care quality values. Positive descriptions were expressions indicating the absence of symptoms or adverse events as well as the occurrence or increase of general wellbeing and satisfaction. Negative descriptions, in turn, were expressions indicating increase or occurrence of adverse events, patient deterioration, symptoms or dissatisfaction. Neutral descriptions did not contain the aforementioned changes in patient's baseline condition or satisfaction. Table 2 presents the matrix of expressions and descriptions of patient status related to nursing care quality.

**TABLE 2:** Matrix of expressions related to nursing care quality and descriptions of patient status related to nursing care quality

Expressions related to nursing care quality *n* = 1676 (100%)	Subcategory	Descriptions of patient status related to nursing care quality, *n* (%)					
		Positive		Neutral		Negative	
Established expressions related to nursing care quality *n* = 748 (44.6%)	Experience of pain	403	(24.0)	13	(0.8)	246	(14.7)
	Adverse event	8	(0.5)	0	(0.0)	51	(3.0)
	Patient satisfaction	15	(0.9)	3	(0.2)	9	(0.5)
Unestablished expressions related to nursing care quality *n* = 928 (55.4%)	Physical health	226	(13.5)	2	(0.1)	131	(7.8)
	Perceived health	240	(14.3)	36	(2.1)	51	(3.0)
	Psychosocial health	19	(1.1)	135	(8.1)	32	(1.9)
	Functional health	40	(2.4)	1	(0.1)	15	(0.9)

### 3.1 Established expressions related to nursing care quality

**FIGURE 1:**
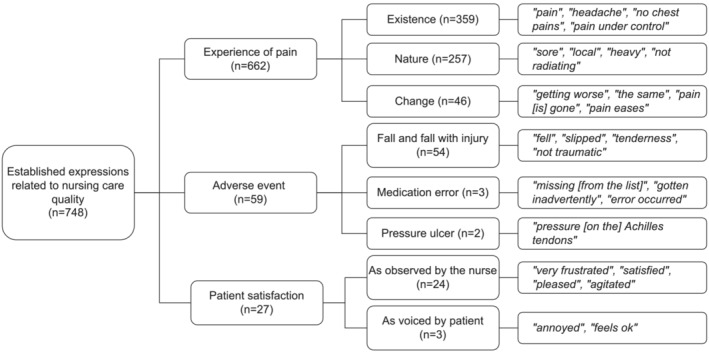
Established expressions related to nursing care quality

Established expressions related to nursing care quality described experiences of pain, adverse events and patient satisfaction, as described in Figure 1.

The subcategory describing patients' *experience of pain* included expressions extracted from free text (*n* = 458, 37.1% of all free text entries) and structured notes (*n* = 204, 46.3% of all structured entries), being eminently the largest category identified with 662 (39.0% of all entries) different expressions. The expressions described the existence, nature and change of experienced pain. Expressions also contained information regarding the location of the pain, such as headache, chest pains, stomach ache or sore throat. The *existence* of pain was described negatively as experiencing (*n* = 129) or positively as not experiencing (*n* = 219) pain in free text notes. Neutral descriptions (*n* = 11) of existence indicated the patient having the pain under control, but also contained diagnostic entries extracted from the structured notes. The *nature* of pain included expressions describing tenderness, soreness, location or radiance of pain, but also the intensity of pain, indicated verbally and using pain rating metrics, such as the Visual Analogue Scale. A total of 197 expressions describing the nature of the experience of pain were extracted from structured notes (44.6% of structured entries), containing numerical information of pain during rest or motion. The expressions related to a *change* in pain perception described an increase or persistence of pain (*n* = 18), but also a decrease or discontinuation of pain (*n* = 27).

The subcategory regarding adverse events (*n* = 59, 4.0% of all) was mainly described in free text (*n* = 58, 4.7% of free text entries). Patient *falls and fall related injuries* described both the occurrence of the fall and details regarding the injury. All these expressions were extracted from free text notes (*n* = 54, 4.4% of free text entries). Expressions describing the occurrence (*n* = 36) were negative verbs indicating the patient falling or slipping during hospitalization. Patient fall related injuries contained information on both the obtaining (*n* = 11) but also the avoidance (*n* = 7) of bruises, dints, tears or undefined injuries. *Medication errors* were among the smallest subcategories with merely three expressions describing occurred errors in the medication process extracted from free text notes. These expressions were all negative, describing defects both in the administration and the documentation of the medication process. *Pressure injuries* were the smallest subcategory with two expressions describing tissue integrity with one expression being extracted from structured and the other from free text notes.

All expressions in the subcategory *patient satisfaction* (*n* = 27, 4.0% of all) were extracted from free text notes (2.2% of free text entries). They were expressions *as described by the nurse* or *as voiced by the patient*. They presented negative descriptions (*n* = 9) of patients being annoyed, frustrated, confused, resentful or agitated, but also neutral expressions (*n* = 3) of being pro‐treatment and positive descriptions (*n* = 15) of being generally pleased.

### 3.2 Unestablished expressions related to nursing care quality

**FIGURE 2:**
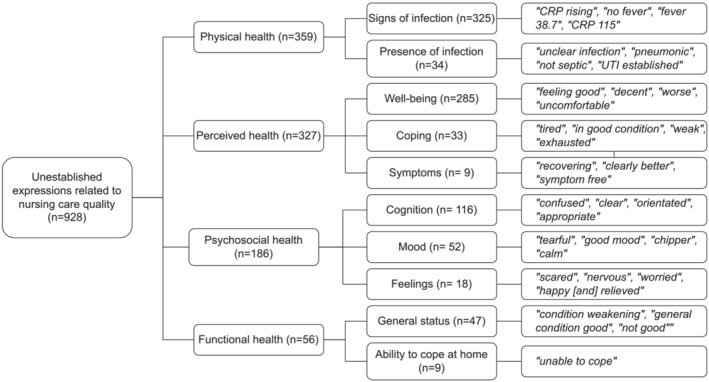
Unestablished expressions related to nursing care quality

Unestablished expressions related to the nursing care quality presented patients' physical, perceived, functional and psychosocial health, as described in Figure 2.

The subcategory regarding patients' *physical health* (*n* = 359, 21.0% of all) contained expressions extracted both from structured (*n* = 235, 53.3% of structured entries) and free text (*n* = 124, 10.0% of free text entries) notes. *Signs of infection* were predominantly extracted from structured notes (*n* = 233, 52.8% of structured entries). They contained information on a patient's body temperature, but also laboratory results on bacterial colonization of wounds, blood and urine as well as C‐reactive protein (CRP), a common indicator used for detecting inflammation from blood. Expressions extracted from free text notes also described elevations and decreases of CRP, patient's body temperature and fever. The *presence of infection* contained expressions indicating the presence (*n* = 21) or absence (*n* = 11) of an undefined infection, symptoms related to an infection, wound infection, hospital acquired infection or sepsis. Neutral expressions (*n* = 2) were diagnostic entries extracted from structured notes. All expressions related to patients' physical health were connected to hospital acquired infections, but only one straight mention to an infections origin as hospital acquired was extracted, with the rest being merely context related.

Expressions in the subcategory covering expressions describing the patients' *perceived health* (*n* = 327, 20% of all) were all extracted from free text notes (26.5% of all free text entries) of which nearly 75% (*n* = 240) were positive descriptions. A patient's perceived health was related to his or her well‐being, coping and symptoms. Positive descriptions (*n* = 237) of *well‐being* included improvement in a patient's condition and generally feeling well. Neutral expressions (*n* = 30) described the patient feeling moderate or normal. Expressions describing the patient feeling ill or getting worse were perceived as negative (*n* = 18). Approximately 90% (*n* = 30) of the expressions describing *coping* were negative descriptions of patient feeling out of strength, exhausted, tired or weary. *Symptoms*, in turn, described mainly neutrally (*n* = 5) with the patient having no symptoms or positively (*n* = 3) as the decrease of symptoms.

The subcategory regarding the patients' *psychosocial health* (*n* = 186, 11.0% of all) included expressions of a patient's cognition, mood and feelings extracted from free text notes (15.0% of all free text entries) except for one structured entry. *Cognition* was generally described with neutral expressions (*n* = 114) of the patient behaving appropriately or being oriented to time and place. No positive expressions describing a patient's cognition were identified. Expressions about *mood* included positive descriptions (*n* = 18) of the patient being in a good mood and feeling high‐spirited to negative descriptions (*n* = 14) of the patient being tearful or seeming stressed, anxious, or feeling low. An expression extracted from structured notes presented a scored depression scale. *Feelings* were described as mainly negative (*n* = 16), with the patient expressing agitation, fear, sadness and worry.

The subcategory of expressions describing patients' *functional health* (*n* = 56, 3.0% of all) were related to a patient's general status and ability to cope at home. Expressions describing the *general status* of patients were mainly perceived as good with positive descriptions (*n* = 40). In contrast, all descriptions (*n* = 9) regarding *patients' abilities to cope at home* were negative. All expressions describing patients' functional health originated from free text notes (4.5% of all free text entries).

## 4 DISCUSSION

The results present an overview of EHR‐based information related to nursing care quality from three differing standpoints that need to be considered in the development and implementation of real‐time information systems for care quality assessment. These include (1) a division between established and unestablished expressions, (2) a presentation of extracted information through their informational value and (3) an acknowledgement of the potential of establishing expressions of different structure in EHRs (i.e., unstructured free text and structured data).

The first standpoint indicates the degree of readiness of nursing‐sensitive indicators to be used in care quality assessment. Previous research presents ample evidence of the use of nursing‐sensitive indicators corresponding to the established expressions found in our study, such as experiences of pain or prevalence of pressure injuries (Seaman et al., 2017). These examples also include research focusing on extracting information related to pain management, ranging from risk identification to the evaluation of analgesic effect or pain prevalence (Nomura et al., 2021). In contrast, before their utilization in systems that help evaluate care quality, more research is needed on unestablished expressions, which are more complicated to interpret. Careful consideration is warranted on how to use these expressions in the assessment care quality. An ample example is the sub‐category describing a patient's physical health containing information on signs and presence of infection. During manual data extraction, single expressions were identified describing hospital acquired infections, a widely accepted nursing quality indicator as presented by Montalvo (2007). Instead of investigating single expressions related to infections as indicating a hospital acquired infection, the timeline and the prevalence of the symptoms must be taken into consideration (Warner et al., 2013). Assessing care quality is perhaps not merely about following single indicators, but more about merging several indicators and examining trends or patterns.

These issues become even more relevant when considering the transition from manual to automated extraction methods. In a study by Ehrentraut et al. (2018), using machine learning methods, the detection of hospital‐acquired infections from structured and free text EHR's showed promising results when comparing records from patients diagnosed with a hospital‐acquired infection to records from undiagnosed patients. These results indicate the potential of text‐classification techniques in real‐world applications, reducing the manual data entry labour of health professionals (Ehrentraut et al., 2018.)

The second standpoint was the presentation of information extracted through their informational value, as illustrated by the presentation of positive, neutral and negative expressions related to nursing care quality. This approach was selected to highlight that nursing care quality is not just the prevalence of absence of negative adverse events. However, in addition to reflecting the nursing care quality, the expressions also reflect the quality of nursing documentation. In a study investigating nurse prioritization leading to unfinished care, documentation was rated among the middle‐ranking tasks (Palese et al., 2020), indicating that during an intense shift some interventions or observations face the danger of being left undocumented. This can lead to issues regarding data accuracy, completeness, and consistency as well as credibility and timeliness (Feder, 2018). When using EHRs as a data source for real‐time care quality assessment, these deficiencies must be understood and taken into consideration from the development phase to end user implementation and beyond. Decision making based on information derived from EHRs requires knowledge not only on the quality indicators used, but also on how the information should be interpreted. Nurse managers' competence requirements should therefore not only address quality assessment and improvement, but also comprehensive expertise on information management. In this study, efforts were made to meet the specific information needs of nurse managers in cardiac care units. However, information on care quality would benefit not only the day‐to‐day management, but also management on other decision‐making levels in health care organizations, providing important bottom‐up information to support upper management as well. From a patient's perspective, care is a continuum of contacts with varying professionals in different settings in the health care system. To form an overall picture, it would be beneficial to acquire quality information from all points of the patient's care pathway (Hanefeld et al., 2017). Expanding future research to explore the needs in other health care settings would benefit both nursing management and patient care.

The importance of promoting guidelines for high quality nursing documentation is evident. An ill‐fitted documentation system may result in inconsistent descriptions regarding patient care, disabling holistic care quality assessment (Rossi et al., 2022). A focus on the information technology competence of nurses and nurse leaders as well as the development of user‐driven systems is warranted. For example, an interview study investigated nurses' perceptions of the effect that EHRs have on patient safety. The results indicated a relationship between EHR usability issues resulting from poor design or user errors and the threat of quality decline. (Tubaishat, 2019.) Additionally, systems based solely on free text documentation entries have been reported to have issues regarding missing information or challenges in locating relevant information, leading to increased risk of missed nursing care (Longhini et al., 2020). It is well established that the implementation of EHRs have increased the time spent on care documentation; however, evidence suggests that after a suitable learning period, work and information flow may start to show signs of improvement (Baumann et al., 2018). This indicates that increasing nurse competence and resources may improve the benefits of both primary and secondary use of EHR data.

The third standpoint was the introduction of data structure by establishing expressions extracted from structured and free text notes. Nearly 75% of all expressions in this study were extracted from free text notes. Structured notes offered no content for example of key adverse events such as medication administration errors or patient falls, nor did they contain relevant information regarding the patients' perceived, psychosocial or functional health. Additionally, the results demonstrate a partial overlap in structured and free text documentation, indicating a burden of multiple entries that increase the professionals' workload. Expressions regarding patients' physical health, for example, presented a multitude of overlaps in expressions related to CRP levels, fever and body temperature with 257 structured entries compared with 458 free text entries, whereas pain intensity was frequently expressed both as numerical structured entries (*n* = 204) as well as free text nurse evaluations and numerical patient evaluations (*n* = 458). The study's results were in line with previous research stating that as a data source, free text notes offer invaluable information related to nursing care quality not available in structured notes (Seaman et al., 2017). Automated data extraction methods would therefore highly benefit from the introduction of NLP to support the secondary use of EHR data.

So far, NLP has been used to identify single nursing‐sensitive indicators such as patient falls from EHRs (Tohira et al., 2021), indicating a possibility for a holistic care quality assessment of using multiple nursing‐sensitive indicators simultaneously, providing reliable and real‐time information to support nurse managers in quality evaluation and improvement initiatives. However, reliable evidence on how these tools function in practice warrants still more research (von Gerich et al., 2022), stressing the importance of not only developing and testing these tools, but also implementing and evaluating them in practice. The results of our study indicate that developing an NLP algorithm for care quality assessment could be used to identify information related to a patient's experience of pain, perceived health, psychosocial health and physical health from EHRs. More research is still needed on how to acquire comprehensive information on adverse events, patient satisfaction or functional health. One question to be resolved is whether the data gap could be narrowed merely by increasing nurse education on documentation, or if additional data sources are needed to provide this information.

The study's limitations are related to the manual data extraction method used in this study, as the expressions were extracted by only one researcher. To increase reliability, the results were regularly reviewed together with another researcher and their evaluation and interpretation discussed. Other limitations include secondary use of EHR's as a data source, as the accuracy and quality of the data are highly dependent on clinical evaluation as well as resources available for documentation.

## 5 CONCLUSIONS

EHRs are a potential data source for assessing care quality, but more research is needed about interpreting and using this information in quality improvement. Issues regarding quality of nursing documentation would benefit from guidelines that promote high‐quality nursing documentation and user‐driven systems, but also from improving the information technology competence of the nurses and nurse management. Entries made in both structured and free text notes increase the risk of double entries, with free text providing a more holistic view on nursing care quality. Assessment care quality could benefit from the introduction of NLP in free text notes. More research is still needed to develop and test such tools in clinical practice.

## 6 IMPLICATIONS FOR NURSING MANAGEMENT

Nurse managers have an essential role in assessing care quality and quality improvement initiatives. The health system generates massive amounts of data continuously, but the tools to effectively utilize this big data for improved data access and interpretation to better support nursing management are lacking. The development, implementation and evaluation of advanced information systems based on user needs would highly benefit knowledge‐based management and support nursing management in quality assessment, by proxy benefiting patient and staff outcomes. They could also provide valuable real time information of the impact of transformational periods, such as the implementation of new technologies, educational interventions and clinical processes. Additionally, using analytical techniques to support nursing tasks is proven to have an impact on the multifaceted and difficult problem of making nursing practise visible (Macieira et al., 2018). In addition to making nursing more visible and providing information to advance the day‐to‐day operations management, systematic automated quality assessment methods could be beneficial to long‐term management as well, presenting information on quality variations over time and revealing trends on a larger scale. This information may be used for benchmarking nursing care quality to monitor performance when compared to others, which could help attract and retain the workforce needed.

## CONFLICT OF INTEREST

None.

## ETHICS STATEMENT

The project held an ethical approval statement (number: 9/2020) issued by University of Turku Ethics Committee for Human sciences (Health Care Division).

## Data Availability

Data sharing not applicable to this article as no datasets were generated or analysed during the current study.
